# Development and validation of a new prognostic immune–inflammatory–nutritional score for predicting outcomes after curative resection for intrahepatic cholangiocarcinoma: A multicenter study

**DOI:** 10.3389/fimmu.2023.1165510

**Published:** 2023-03-31

**Authors:** Jiang Zhu, Denghui Wang, Chang Liu, Rui Huang, Fengwei Gao, Xuping Feng, Tian Lan, Hui Li, Hong Wu

**Affiliations:** ^1^ Liver Transplantation Center, West China Hospital, Sichuan University, Chengdu, China; ^2^ Department of Liver Surgery, West China Hospital, Sichuan University, Chengdu, China; ^3^ Department of Hepatopancreatobiliary Minimal Invasive Surgery, Chengdu ShangJin NanFu Hospital, Chengdu, China; ^4^ Department of Hepatobiliary Pancreatic Tumor Center, Chongqing Key Laboratory of Translational Research for Cancer Metastasis and Individualized Treatment, Chongqing University Cancer Hospital, Chongqing, China; ^5^ Department of Hepatobiliary Surgery, The People's Hospital of Leshan, Leshan, China; ^6^ Department of Anesthesiology, The Second Affiliated Hospital, Zhejiang University School of Medicine, Hangzhou, China; ^7^ Department of Endocrine and Breast Surgery, The First Affiliated Hospital of Chongqing Medical University, Chongqing, China

**Keywords:** intrahepatic cholangiocarcinoma, immunity, inflammation, nutrition, prognosis, nomogram

## Abstract

**Background:**

Immune function, nutrition status, and inflammation influence tumor initiation and progression. This was a retrospective multicenter cohort study that investigated the prognostic value and clinical relevance of immune-, inflammatory-, and nutritional-related biomarkers to develop a novel prognostic immune–inflammatory–nutritional score (PIIN score) for patients with intrahepatic cholangiocarcinoma (ICC).

**Methods:**

The clinical data of 571 patients (406 in the training set and 165 in the validation set) were collected from four large hepato-pancreatico-biliary centers of patients with ICC who underwent surgical resection between January 2011 and September 2017. Twelve blood biomarkers were collected to develop the PIIN score using the LASSO Cox regression model. The predictive value was further assessed using validation datasets. Afterward, nomograms combining the PIIN score and other clinicopathological parameters were developed and validated based on the calibration curve, time-dependent AUC curves, and decision curve analysis (DCA). The primary outcomes evaluated were overall survival (OS) and recurrence-free survival (RFS) from the day of primary resection of ICC.

**Results:**

Based on the albumin–bilirubin (ALBI) grade, neutrophil- to- lymphocyte ratio (NLR), prognostic nutritional index (PNI), and systemic immune- inflammation index (SII) biomarkers, the PIIN score that classified patients into high-risk and low-risk groups could be calculated. Patients with high-risk scores had shorter OS (training set, p < 0.001; validation set, p = 0.003) and RFS (training set, p < 0.001; validation set, p = 0.002) than patients with low-risk scores. The high PIIN score was also associated with larger tumors (≥5 cm), lymph node metastasis (N1 stage), multiple tumors, and high tumor grade or TNM (tumor (T), nodes (N), and metastases (M)) stage. Furthermore, the high PIIN score was a significant independent prognostic factor of OS and RFS in both the training (p < 0.001) and validation (p = 0.003) cohorts, respectively. A PIIN-nomogram for individualized prognostic prediction was constructed by integrating the PIIN score with the clinicopathological variables that yielded better predictive performance than the TNM stage.

**Conclusion:**

The PIIN score, a novel immune–inflammatory–nutritional-related prognostic biomarker, predicts the prognosis in patients with resected ICC and can be a reliable tool for ICC prognosis prediction after surgery. Our study findings provide novel insights into the role of cancer-related immune disorders, inflammation, and malnutrition.

## Introduction

Intrahepatic cholangiocarcinoma (ICC) is the second most prevalent primary solid tumor of the liver after hepatocellular carcinoma, with an increasing incidence worldwide over the past few decades (1, 2). Currently, the efficacy of the available treatment options for ICC, including chemotherapy, targeted therapy, and immunotherapy, is limited; surgical resection remains the only curative method (3). Nevertheless, the postoperative recurrence rate is up to 80%, and the 5-year overall survival (OS) of ICC patients is less than 20% (4, 5). Several ordinary clinicopathologic characteristics, such as tumor size, histological grade (6), multifocality (7), lymph node metastasis (LNM) (8, 9), microvascular invasion (MVI) (10), and carbohydrate antigen 19-9 (CA19-9) (11), are used for prognosis evaluation and risk stratification in patients with ICC. However, the value of these conventional factors in predicting ICC prognosis is limited. Novel accurate prognostic indicators in patients with ICC are urgently needed. Recent studies have shown that immune function, nutrition status, and inflammation participate in tumor initiation and progression (12–17). Several immune-, inflammatory-, and nutritional-related biomarkers based on preoperative blood indexes are valuable prognostic markers in many cancer types (18–21). Previous studies have demonstrated that a low controlling nutritional status (CONUT) score and prognostic nutritional index (PNI) score are associated with favorable prognosis, and an elevated systemic immune inflammation index (SII) level worsens OS in patients with ICC (22–24). However, a single blood marker cannot reflect the landscape of a patient’s immune function, nutrition status, and inflammation. It remains unclear whether this combination may help to overcome this limitation. This study analyzed the prognostic role and clinical relevance of immune-, inflammatory-, and nutritional-related factors to develop a novel predictive model termed the prognostic immune–inflammatory–nutritional score (PIIN score). The PIIN score combines immune-, inflammatory-, and nutritional-related biomarkers to evaluate the outcomes of ICC resection. Furthermore, PIIN-nomograms based on the PIIN score and other clinicopathological features were constructed and validated for individualized predictions of the survival probability of the patients.

## Materials and methods

### Study cohort

Patients with ICC undergoing curative resection between January 2011 and September 2017 at West China Hospital, Chongqing University Cancer Hospital, the People’s Hospital of Leshan, and Chengdu Shang Jin Nan Fu Hospital were screened. The inclusion criteria were as follows: (1) patients with histologically proven ICC; (2) with radical resection; (3) with no extrahepatic metastasis; (4) with complete baseline laboratory test information. The exclusion criteria were as follows: (1) patients who received other treatments before surgery (transarterial chemoembolization, radiofrequency ablation, or systemic therapy); (2) with other malignancy histories; (3) with missing clinical data or follow-up information. The included patients were randomly divided into training (n = 406) and validation sets (n = 165) at a ratio of 7:3.

### Data collection and definition of variables

The demographic and tumor-related characteristics comprised the following: age, sex, tumor diameter, tumor number, hepatolithiasis, perineural invasion, biliary invasion, histological grade, MVI, lymph node status, and the 8th edition of the American Joint Committee on Cancer (AJCC)-TNM classification. Preoperative hematological parameters were evaluated; these included fasting blood glucose (FBG); lymphocyte, neutrophil, and platelet (PLT) counts; fibrinogen (FIB); alanine aminotransferase (ALT); aspartate aminotransferase (AST); bilirubin; albumin; globulin; gamma-glutamyl transpeptidase (GGT); cholesterol; alkaline phosphatase (ALP); and carbohydrate antigen 19-9 (CA19-9) levels. This study focused on 12 immune-, inflammatory-, and nutritional-related biomarkers: ALT, AST, albumin–alkaline phosphatase ratio (AAPR), albumin–globulin ratio (AGR), FIB, albumin–bilirubin (ALBI) grade, GGT–albumin ratio (GAR), neutrophil- to- lymphocyte ratio (NLR), platelet- to- lymphocyte ratio (PLR), prognostic nutritional index (PNI), systemic immune inflammation index (SII), and controlling nutritional status (CONUT). The biomarkers were calculated as follows: AAPR = albumin (g/L)/ALP (IU/L), AGR= albumin (g/L)/globulin (g/L), ALBI= log_10_bilirubin (mol/L) × 0.66–albumin (g/L) ×0.085, GAR= FBG (mmol/L)/lymphocyte, NLR = neutrophil/lymphocyte, PLR = platelet/lymphocyte, PNI = albumin (g/L) + 5 × lymphocyte, and SII = platelet × neutrophil/lymphocyte. The CONUT score was calculated based on the albumin concentration, total lymphocyte count, and total cholesterol concentration (**Supplementary Table 1**). The maximally selected rank determined the appropriate cut-off values of the continuous parameters. The ALBI grade was classified according to cut-off value ≤-2.60 (ALBI grade 1), >-2.60 to ≤-1.39 (ALBI grade 2), and ≥-1.39 (ALBI grade 3), as previously described. The patients were divided into the low and high CONUT score groups based on the median score. Overall survival (OS) was defined as the interval from the date of curative surgery to death and the last follow-up. Recurrence-free survival (RFS) was defined as the time between curative surgery and disease recurrence and the last follow-up.

### PIIN score construction

Briefly, univariate Cox regression analysis was performed to screen prognostic immune-, inflammatory-, and nutritional-related biomarkers. Statistical significance was set at *P*< 0.05 and maintained for further analysis. The prognostic significance of these prognostic-related biomarkers was evaluated using the least absolute shrinkage and selection operator (LASSO) Cox regression analysis. Finally, the prognostic immune–inflammatory–nutritional score (PIIN score) was calculated based on variables without zero coefficients.

### Prognostic value of PIIN score

To investigate whether the PIIN score is associated with adverse clinicopathological characteristics, the PIIN score between the two subgroups of different clinicopathological characteristics was compared using the Wilcoxon test. The predictive value of the PIIN score was evaluated using receiver operating characteristic (ROC) curves for the 1-, 3-, and 5- year OS of the ICC patients in the training and validation sets. The median PIIN score, as a cut-off value, divided patients into high-risk and low-risk groups. Univariate and multivariate Cox regression analyses were performed to identify prognostic factors independently related to ICC prognosis.

### Construction and validation of nomograms

The prognostic nomograms for the survival outcomes (OS and RFS) in the training set were constructed based on multivariate Cox regression analyses by backward stepwise selection with the smallest Akaike information criterion (AIC) value. Time-dependent AUC and calibration curves were used to validate the predictive performance of the nomogram in the training and validation sets. The clinical application values between the nomogram and the AJCC-TNM staging system were compared using decision curve analysis (DCA).

### Statistical analysis

A t-test was used to compare continuous variables between the two groups. The chi-square test was used to analyze the differences between the groups of categorical data. The Kaplan–Meier (K-M) curves were plotted to compare differences in OS and RFS using the log-rank test. Statistical analysis was performed using R version 4.1.3. P < 0.05 was considered statistically significant.

## Results

### Baseline characteristics and PIIN score

**Figure 1:**
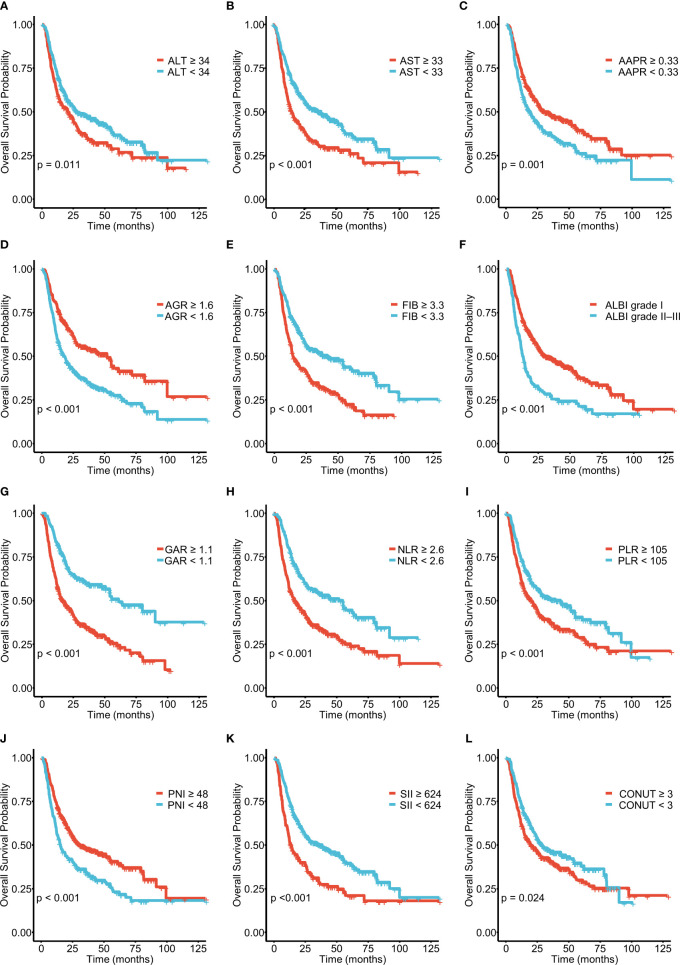
Kaplan–Meier curves for overall survival (OS), stratified by **(A)** ALT, **(B)** AST, **(C)** AAPR, **(D)** AGR, **(E)** FIB, and **(F)** ALBI grades and **(G)** GAR, **(H)** NLR, **(I)** PLR, **(J)** PNI, **(K)** SII, and **(L)** CONUT scores in patients with ICC. ICC, intrahepatic cholangiocarcinoma; ALT, alanine aminotransferase; AST, aspartate aminotransferase; AAPR, albumin–alkaline phosphatase ratio; AGR, albumin–globulin ratio; ALBI, albumin–bilirubin grade; FIB, fibrinogen; GAR, GGT–albumin ratio; NLR, neutrophil- to- lymphocyte ratio; PLR, platelet-to-lymphocyte ratio; PNI, prognostic nutritional index; SII, systemic immune inflammation index; CONUT, controlling nutritional status.

**Figure 2:**
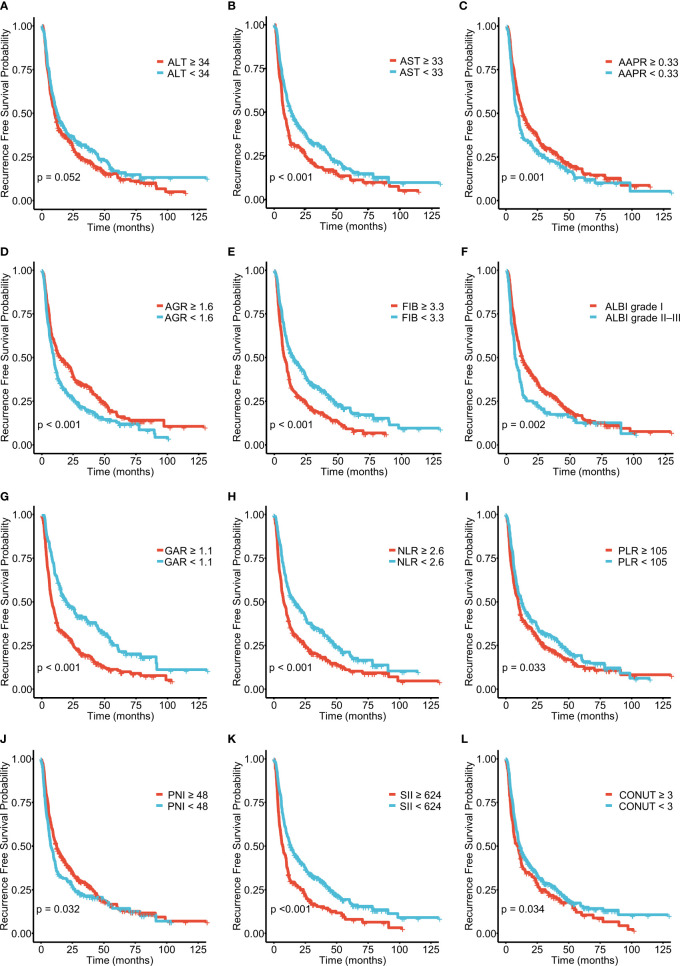
Kaplan–Meier curves for recurrence-free survival (RFS), stratified by **(A)** ALT, **(B)** AST, **(C)** AAPR, **(D)** AGR, **(E)** FIB, and **(F)** ALBI grades and **(G)** GAR, **(H)** NLR, **(I)** PLR, **(J)** PNI, **(K)** SII, and **(L)** PLR in patients with ICC.

**Figure 3:**
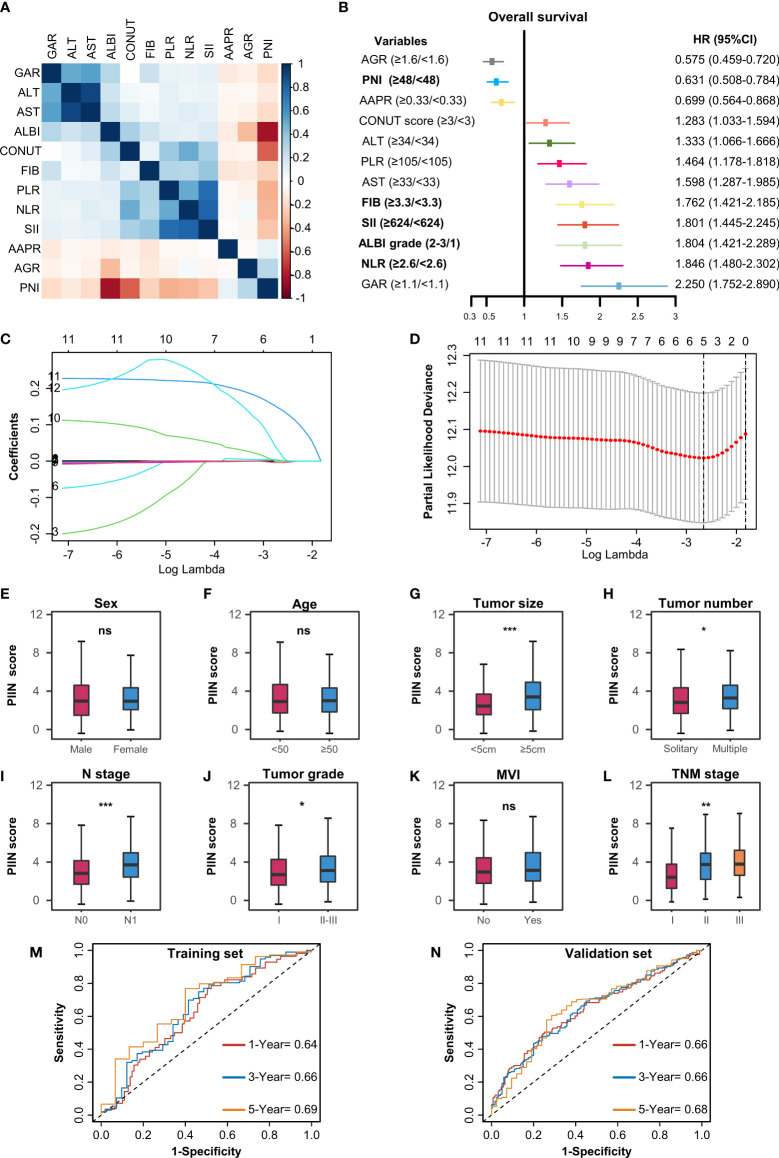
Construction of the PIIN score using the LASSO Cox regression model. **(A)** Heatmap of the correlations of the immune–inflammatory–nutritional- related biomarkers. **(B)** Forest plot of the univariate Cox regression analysis for OS. **(C)** Partial likelihood deviance for LASSO coefficient profiles. The red dots represent the partial likelihood values, the grey lines represent the standard error (SE), and the vertical dotted line shows the optimal values by 1-s.e. **(D)** Least absolute shrinkage and selection operator (LASSO) coefficient profiles of 12 immune–inflammatory–nutritional- related biomarkers. **(E-L)** Differential analysis of the distribution of the PIIN scores in different clinicopathologic features. A comparison between the two groups was performed using the Wilcoxon test. Three group comparisons were performed using the Kruskal–Wallis test. *P< 0.05; **P< 0.01; ***P< 0.001; ns not significant. The ROC curves for predicting OS at 1-, 3-, and 5 years in the training set **(M)** and the validation set **(N)**. ROC, receiver operating characteristic.

**Figure 4:**
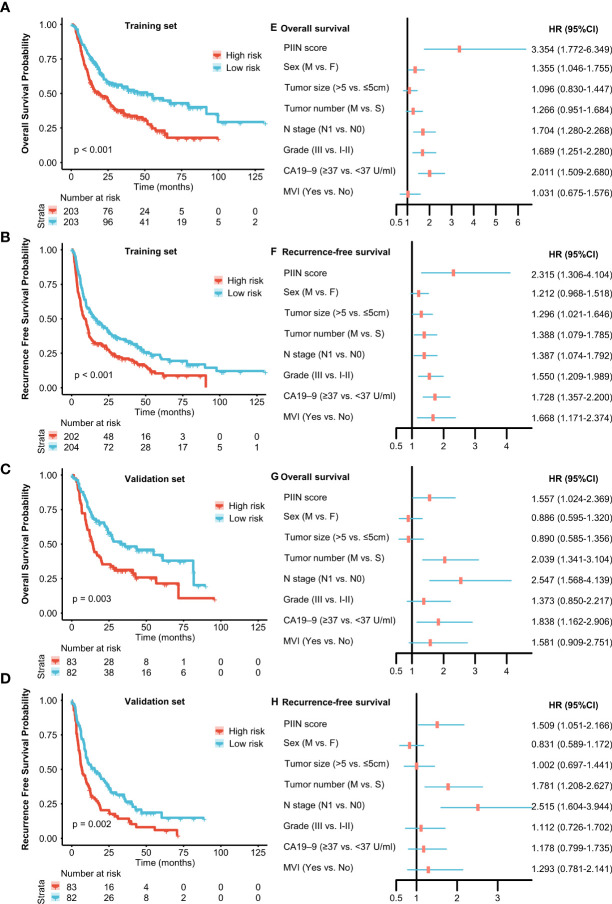
Prognostic implications of the PIIN score. Kaplan–Meier curves of OS **(A)** and RFS **(B)** for patients in the low- and high-risk groups according to the PIIN score in the training set. Kaplan–Meier curves of OS **(C)** and RFS **(D)** for patients in the low- and high-risk groups according to the PIIN score in the validation set. Forest plot of multivariable Cox regression analysis of OS **(E)** and RFS **(F)** in the training set. Forest plot of multivariable Cox regression analysis of OS **(G)** and RFS **(H)** in the validation set.

A total of 571 patients were included in this study, of whom 49.6% were male and 50.4% female. Their median age was 59 years (range 50–65 years), and the median follow-up time was 30 months (range 3–126 months). The general population’s 1-, 3-, and 5-year OS were 74.1%, 37.3%, and 21.8%, respectively. The baseline demographic and clinical characteristics of the training (n = 406) and validation cohorts (n = 165) are presented in **Table 1**. The distribution of these baseline variables in the two cohorts was well-balanced (p>0.05). The Kaplan–Meier survival analysis revealed low ALT, AST, ALBI grades, low FIB, GAR, NLR, PLR, SII, and CONUT scores, and high AAPR, AGR, and PNI, which were significantly associated with improved OS (**Figure 1**). Similarly, the Kaplan–Meier analysis for RFS was consistent (P< 0.05) (**Figure 2**). The correlations of the 12 immune-, inflammatory-, and nutritional-related biomarkers are shown in **Figure 3A**. Univariate Cox regression analysis showed that these biomarkers were associated with OS (p< 0.05, **Figure 3B**). To determine the independent prognostic biomarkers, all the variables mentioned above were included in the LASSO Cox regression analysis. The LASSO Cox regression analysis revealed that only four biomarkers, including SII, NLR, PNI, and ALBI, with non-zero coefficients were found to be associated with ICC prognosis after surgery (**Figure 3C**). The PIIN score was constructed using the formula: Riskscore=NLR*0.876+SII*0.0174+FIB*14.355+ALBI*2.209- PNI*0.386. First, the relationship between the PIIN score and clinicopathological characteristics was assessed. The patients with larger tumors (≥5 cm), lymph node metastasis (N1 stage), multiple tumors, and a higher tumor grade or TNM stage had a higher PIIN score. No association between age, sex, MVI, and PIIN score was found (**Figures 3E-L**), suggesting that the PIIN score is only associated with adverse clinical and tumoral characteristics. The ROC analysis enhanced the accuracy of the PIIN score, with 1-, 3-, and 5-year OS for AUCs of 0.64,0.66, and 0.69 in the training set (**Figure 3M**) and 0.66, 0.66, and 0.68 in the validation set, respectively (**Figure 3N**). The ROC value of the PIIN score was significantly higher than that of a single blood marker in the training and validation cohorts (**Supplemental Figure 1**). Subsequently, the patients with ICC were classified into low- and high-risk groups based on the medium cut-off value of 3.7. The Kaplan–Meier analysis suggested that patients in the high-risk group had shorter OS (training set, p < 0.001; validation set, p = 0.003) and RFS (training set, p < 0.001; validation set, p = 0.002) than those in the low-risk group (**Figures 4A-D**). The results of the univariate Cox regression analysis are shown in **Table 2**. After adjusting for other clinicopathologic factors, multivariate Cox regression analysis of the patients in the training set revealed that the PIIN score was one of the independent prognostic factors of survival outcomes of the ICC patients (OS: HR 0.89, 95% CI 0.84–0.94; RFS: HR 0.88, 95% CI 0.83–0.94) (**Figures 4E, F**). In the validation cohort, the PIIN score was similar for OS and RFS (HR 0.89, 95% CI 0.84–0.94) (**Figures 4G, H**). These results demonstrate that the PIIN score is vital and may be an independent predictor of ICC prognosis after resection.

**Table 1:** Comparison of clinicopathological characteristics in training and validation sets.

Variables	All patients (N = 571)	Training set (N=406)	Validation set (N=165)	P value
Age (year) , median [IQR]	59 [50–65]	58 [50–65]	60.0 [52–66]	0.213
Sex				0.814
Male	283 (49.6%)	203 (50.0%)	80 (48.5%)	
Female	288 (50.4%)	203 (50.0%)	85 (51.5%)	
Tumor size (cm) , median [IQR]	5.6 [4.0–7.7]	5.7 [4.2–8.0]	5.6 [4.0–7.5]	0.615
Tumor number				0.678
Single	417 (73.0%)	299 (73.6%)	118 (71.5%)	
Multiple	154 (27.0%)	107 (26.4%)	47 (28.5%)	
Grade				0.827
III	382 (66.9%)	270 (66.5%)	112 (67.9%)	
I-II	189 (33.1%)	136 (33.5%)	53 (32.1%)	
Hepatolithiasis				0.729
No	467 (81.8%)	334 (82.3%)	133 (80.6%)	
Yes	104 (18.2%)	72 (17.7%)	32 (19.4%)	
Perineural invasion				0.842
No	482 (84.4%)	344 (84.7%)	138 (83.6%)	
Yes	89 (15.6%)	62 (15.3%)	27 (16.4%)	
Microvascular invasion				0.210
No	511 (89.5%)	368 (90.6%)	143 (86.7%)	
Yes	60 (10.5%)	38 (9.36%)	22 (13.3%)	
Biliary invasion				0.311
No	509 (89.1%)	358 (88.2%)	151 (91.5%)	
Yes	62 (10.9%)	48 (11.8%)	14 (8.48%)	
Lymph node metastasis				0.652
No	434 (76.0%)	306 (75.4%)	128 (77.6%)	
Yes	137 (24.0%)	100 (24.6%)	37 (22.4%)	
CA19–9				0.020
≥37 U/ml	349 (61.1%)	235 (57.9%)	114 (69.1%)	
<37 U/ml	222 (38.9%)	171 (42.1%)	51 (30.9%)	
TNM stage				
I–II	161 (28.2%)	115 (28.3%)	46 (27.9%)	0.996
III	410 (71.8%)	291 (71.7%)	119 (72.1%)	
CONUT score				0.819
<3	257 (45.0%)	181 (44.6%)	76 (46.1%)	
≥3	314 (55.0%)	225 (55.4%)	89 (53.9%)	
ALBI grade				0.128
1	449 (78.6%)	312 (76.8%)	137 (83.0%)	
2–3	122 (21.4%)	94 (23.2%)	28 (17.0%)	
ALT (IU/L) , median [IQR]	25 [16–41]	26 [17–41]	23 [16–39]	0.344
AST (IU/L) , median [IQR]	29 [22–38]	29 [23–38]	29 [21–38]	0.485
AAPR, median [IQR]	0.38 [0.27–0.51]	0.38 [0.26–0.52]	0.37 [0.29–0.51]	0.862
AGR, median [IQR]	1.51 [1.30–1.70]	1.50 [1.27–1.68]	1.53 [1.37–1.75]	0.032
FIB (g/L), median [IQR]	3.22 [2.56–3.93]	3.22 [2.55–3.95]	3.23 [2.70–3.89]	0.997
Glu (mmol/L), median [IQR]	5.13 [4.66–5.80]	5.09 [4.66–5.68]	5.22 [4.70–5.87]	0.263
GAR, median [IQR]	1.68 [0.81–3.67]	1.62 [0.80–3.43]	1.78 [0.83–4.53]	0.233
GGT (IU/L) , median [IQR]	71 [35–146]	68 [34–138]	81 [36–174]	0.152
NLR, median [IQR]	2.72 [1.95–3.93]	2.73 [1.95–3.84]	2.64 [1.96–3.94]	0.941
PLR, median [IQR]	111 [82–152]	113 [83–152]	107 [80–150]	0.806
PNI, median [IQR]	50.8 [46.9–53.4]	50.5 [46.8–53.1]	51.0 [47.2–53.8]	0.171
SII, median [IQR]	451 [288–749]	448 [289–746]	487 [291–755]	0.582

IQR, interquartile ranges; ALBI, albumin–bilirubin; ALT, alanine aminotransferase; AST, aspartate aminotransferase; AAPR, album-alkaline phosphatase ratio; AGR, albumin-to-globulin ratio; CA19-9, cancer antigen 19-9; CONUT score, controlling nutritional status score; FIB, fibrinogen; GGT, gamma-glutamyl transferase; GAR, gamma-glutamyl transferase to albumin ratio; NLR, neutrophil-lymphocyte ratio; PLR, platelet-to-lymphocyte ratio; PNI, prognostic nutritional index; SII, systemic immune-inflammation index.

**Table 2:** Results of univariate survival analysis in training and validation sets.

Univariate Cox Regression analysis for OS				
Variables	Training set		Validation set	
	HR (95%CI)	P value	HR (95%CI)	P value
PIIN score	5.847 (3.187–10.730)	< 0.001	4.636 (1.941–11.072)	< 0.001
Sex (M vs. F)	1.284 (0.993–1.661)	0.062	0.912 (0.619–1.343)	0.648
Age (>60 vs. ≤60)	1.017 (0.787–1.315)	0.897	1.146 (0.780–1.684)	0.486
Tumor size (>5 vs. ≤5cm)	1.335 (1.025–1.738)	< 0.001	1.152 (0.780–1.701)	0.457
Tumor number (Multiple vs. Solitary)	1.437 (1.088–1.899)	0.011	2.214 (1.478–3.316)	< 0.001
N stage (N1 vs. N0)	2.213 (1.680–2.914)	< 0.001	3.423 (2.226–5.263)	< 0.001
TNM stage (III vs. I–II)	1.463 (1.083–1.977)	0.013	1.231 (0.798–1.900)	0.347
Grade (III vs. I-II)	1.887 (1.406–2.532)	< 0.001	1.613 (1.043–2.493)	0.032
CA19–9 (≥37 vs. <37 U/ml)	2.196 (1.661–2.904)	< 0.001	1.646 (1.058–2.563)	0.027
Hepatolithiasis (Yes vs. No)	1.456 (1.062–1.996)	0.019	1.450 (0.924–2.276)	0.106
MVI (Yes vs. No)	1.379 (0.909–2.091)	0.130	2.131 (1.290–3.518)	0.003
Biliary invasion (Yes vs. No)	1.152 (0.793–1.674)	0.457	1.003 (0.486–2.067)	0.994
Perineural invasion (Yes vs. No)	1.411 (1.003–1.986)	0.048	2.610 (1.627–4.187)	< 0.001
**Univariate Cox Regression analysis for RFS**				
PIIN score	4.237 (2.440–7.357)	< 0.001	2.820 (1.315–6.046)	0.008
Sex (M vs. F)	1.163 (0.932–1.452)	0.181	0.862 (0.615–1.209)	0.390
Age (>60 vs. ≤60)	1.106 (0.885–1.383)	0.376	1.198 (0.855–1.677)	0.294
Tumor size (>5 vs. ≤5cm)	1.495 (1.190–1.880)	< 0.001	1.176 (0.835–1.657)	0.353
Tumor number (Multiple vs. Solitary)	1.581 (1.240–2.017)	< 0.001	2.000 (1.375–2.909)	< 0.001
N stage (N1 vs. N0)	1.754 (1.368–2.248)	< 0.001	2.993 (1.997–4.487)	< 0.001
TNM stage (III vs. I–II)	1.321 (1.025–1.702)	0.031	1.854 (1.238–2.777)	< 0.001
Grade (III vs. I-II)	1.649 (1.292–2.105)	< 0.001	1.471 (1.013–2.137)	0.043
CA19–9 (≥37 vs. <37 U/ml)	1.838 (1.457–2.320)	< 0.001	1.153 (0.798–1.665)	0.449
Hepatolithiasis (Yes vs. No)	1.034 (0.776–1.378)	0.822	1.180 (0.768–1.812)	0.449
MVI (Yes vs. No)	1.916 (1.351–2.718)	< 0.001	1.718 (1.064–2.775)	0.027
Biliary invasion (Yes vs. No)	1.101 (0.780–1.553)	0.583	1.126 (0.607–2.089)	0.706
Perineural invasion (Yes vs. No)	1.119 (0.818–1.531)	0.484	2.128 (1.376–3.290)	< 0.001

PIIN, inflammatory-nutritional prognostic scoring; CA-199, cancer antigen 19-9; MVI, microvascular invasion; OS, overall survival; RFS, recurrence-free survival.

### Development and validation of PIIN-nomograms

**Figure 5:**
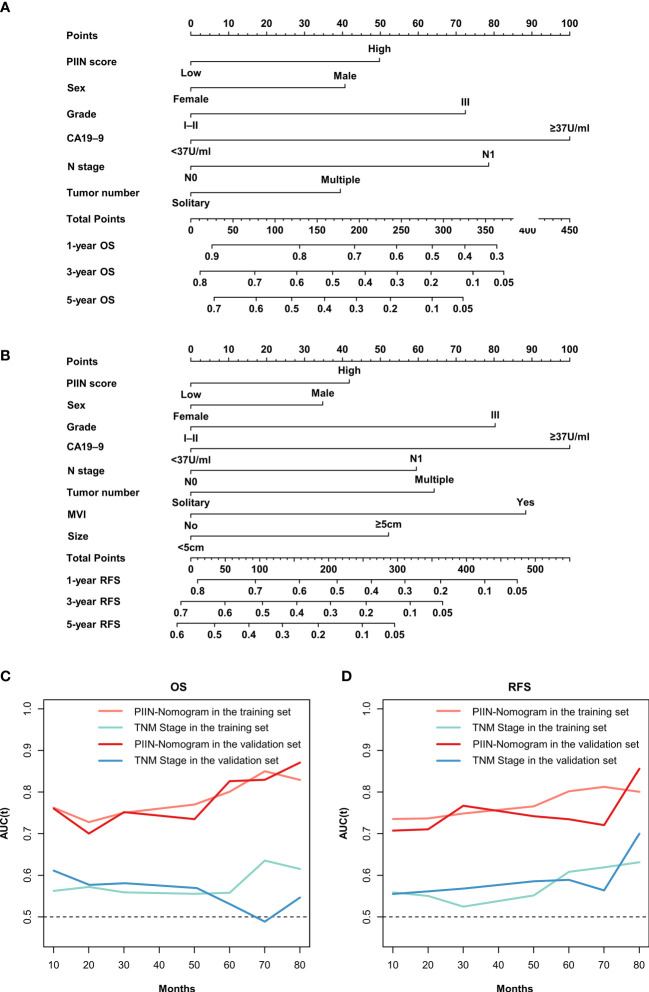
Construction and validation of the nomograms. Nomograms incorporating the PIIN score and other clinicopathological parameters for OS **(A)** and RFS **(B)** prediction in the training cohort. **(C)** Time-dependent AUC curves of the PIIN-nomogram and the AJCC-TNM staging system for the prediction of OS in the training and validation sets. **(D)** Time-dependent AUC curves of the PIIN-nomogram and the AJCC-TNM staging system for predicting RFS in the training and validation sets. AUC, area under the curve; AJCC-TNM, American Joint Committee on Cancer tumor–node–metastasis.

**Figure 6:**
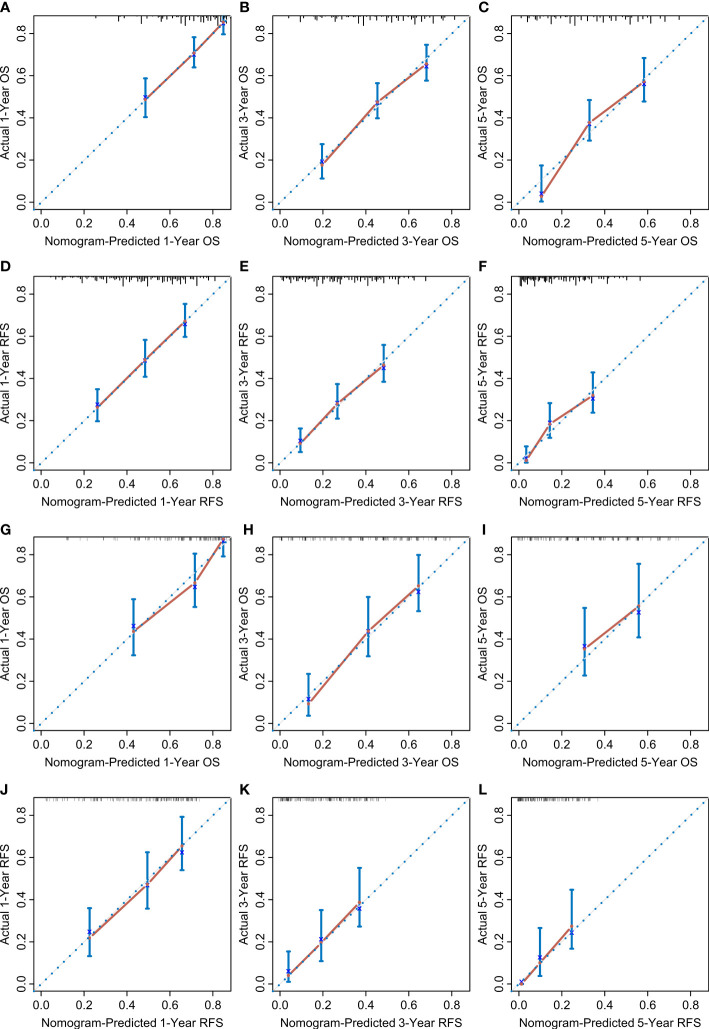
Calibration curves. The calibration curves of the nomograms between predicted and observed 1-, 3-, and 5-year OS of patients in the training set **(A–C)** and the validation set **(G–I)**. The calibration curves of the nomograms between predicted and observed 1-, 3-, and 5-year RFS in the training set **(D–F)** and the validation set **(J–L)**. The dashed line of 45° represents the perfect prediction of the nomogram.

**Figure 7:**
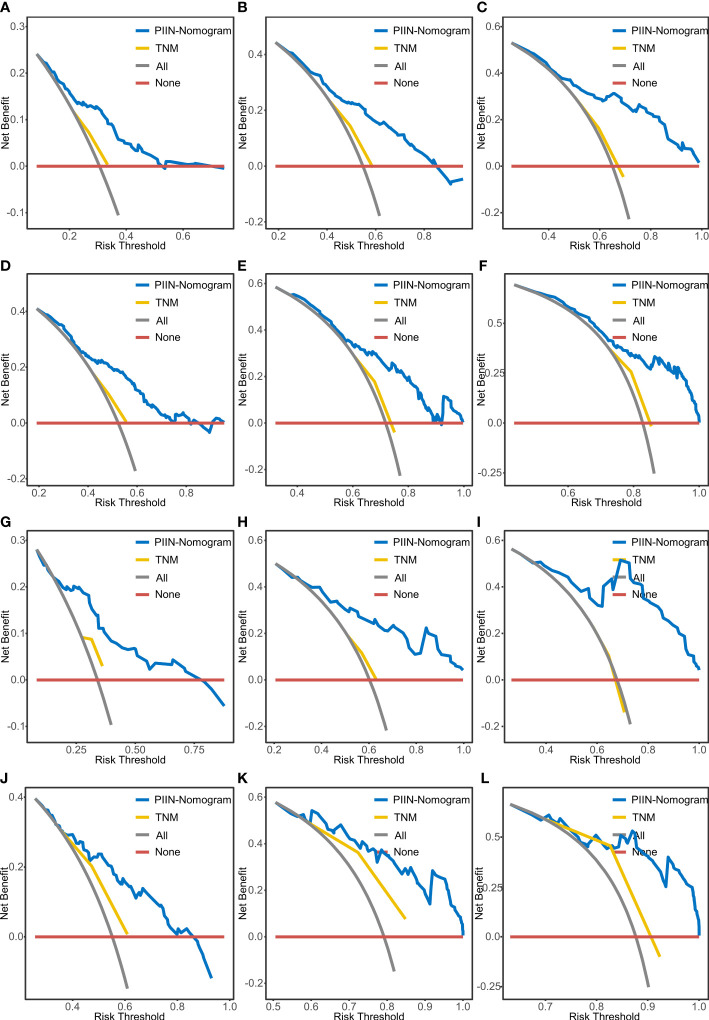
DCA of OS and RFS prediction by the nomograms. The DCA of the nomogram and AJCC-TNM stage for 1-year OS **(A)**, 3-year OS **(B)**, and 5-year OS **(C)** and for 1-year RFS **(D)**, 3-year RFS **(E)**, and 5-year RFS **(F)** in the training set. DCA of the nomogram and AJCC-TNM stage for 1-year OS **(G)**, 3-year OS **(H)**, and 5-year OS **(I)** and for 1-year RFS **(J)**, 3-year RFS **(K)**, and 5-year RFS **(L)** in the validation set. DCA, decision curve analysis; AJCC-TNM, American Joint Committee on Cancer tumor–node–metastasis.

According to the multivariate Cox regression analysis by backward stepwise selection with the smallest AIC value, the PIIN score, sex, grade, CA19-9, N stage, and tumor number were used in the final nomogram for OS (**Figure 5A**). The PIIN score, sex, grade, CA19-9, N stage, MVI, tumor number, and tumor size were included in the nomogram for RFS (**Figure 5B**). Based on their predictive ability, time-dependent AUC curves were plotted to compare the predictive accuracy of the PIIN-nomograms with the AJCC-TNM staging system. The AUC values for OS and RFS nomograms were significantly higher than those of the AJCC-TNM staging system in the training and validation sets (**Figures 5C, D**). The calibration curves also showed consistency between nomogram prediction and observed survival outcomes (**Figure 6**). Collectively, the two PIIN-nomograms were particularly discriminative and calibrative. Furthermore, DCA was performed to evaluate the clinical application of the nomogram by quantifying the net benefits at different threshold probabilities (**Figure 7**). DCA showed that the PIIN-nomograms were superior to the AJCC-TNM staging system in predicting ICC prognosis.

## Discussion

A specific connection exists between immunity, inflammation, nutrition, and cancer (15, 25, 26). Several studies have demonstrated the core role of cancer-related inflammation in cancer development and progression (14, 27). Previous studies revealed that the relevant biomarkers, such as the systemic inflammation score (SIS) and inflammatory marker C-reactive protein (CRP), can predict the prognosis of cancer patients (28, 29). Several nutrition indexes, such as the BMI, PNI, and CONUT scores, have also been reported as potential prognostic cancer predictors (30, 31). Immunity-related biomarkers derived from peripheral blood are significantly associated with survival outcomes in multiple human cancers, including ICC (21, 32–34).

A single indicator is insufficient for prognosis risk stratification, highlighting the urgent need to integrate these markers. Immune-, inflammatory-, and nutritional-related biomarkers were extensively collected in this study based on the peripheral blood testing of patients with ICC. To our knowledge, this is the first study to evaluate the prognostic value and clinical relevance of immune-, inflammatory-, and nutritional-related factors and to establish the PIIN score based on these biomarkers. Our study findings showed that the ALT, AST, and ALBI grades, the AAPR, AGR, FIB, GAR, NLR, PLR, SII, and CONUT scores, and PNI are significantly related to the survival of patients with ICC. The PIIN score was a valuable marker for prognostic stratification and was predictive for patients in the training and validation sets. Moreover, compared with the TNM staging system, nomograms used in this study combined the PIIN score and clinical factors, improving the survival prediction of patients with ICC.

The PIIN score consists of four biomarkers (NLR, SII, FIB, and ALBI grade) reflecting the immune function, inflammation, and nutrition status. The predictive capacity of these markers has been confirmed in several types of cancer. The ALBI grade, proposed by Japanese scholars in 2014 (35), is an independent prognostic predictor after hepatic resection in the early stage of ICC (36), because it is calculated from albumin and bilirubin. A high ALBI grade causes high levels of malnutrition (37) and was associated with worse outcomes in patients with ICC. Consistent with previous reports (38, 39), serum NLR was an independent prognostic factor for the survival of patients with ICC. The increase in serum NLR levels reflected lymphopenia, neutrophilia, or both and represented an inability of the immune system to suppress cancer progression, thus contributing to poor prognosis. SII comprises peripheral blood, lymphocyte, platelet counts, and neutrophil, which can comprehensively estimate the host’s immune response and inflammatory status (18, 23). Hu et al. (40) found that SII is significantly associated with worse survival in hepatocellular carcinoma. Our study findings demonstrate that SII could also serve as a valuable independent prognostic factor for patients with ICC. Moreover, the complex cross-talk between the activation of coagulation and inflammation in cancer has received much attention. Abnormal hypercoagulable condition is often observed in most cancer patients (41). FIB is a glycoprotein in coagulation and is synthesized by the hepatocytes. It promotes tumor development, angiogenesis, and metastasis (42, 43). In this study, FIB was a useful prognostic marker for evaluating clinical outcomes in patients with ICC. The biochemical parameters were routinely examined for preoperative blood tests. Therefore, the PIIN score can be easily obtained in a clinical setting. The favorable predictive performance of the PIIN score implies that it is associated with malnutrition, immunosuppression, active inflammatory reaction, and tumor progression. As validated in the multicenter dataset, the PIIN score was expected to have a considerable predictive capacity for ICC. A nomogram model was constructed to accurately predict the survival of patients with ICC by incorporating the PIIN score and other clinicopathological variables with the time-dependent AUC reaching about 0.70~0.88. This study had several limitations. First, given that all the participants were Chinese patients, the PIIN score in other cohorts worldwide was not evaluated using the nomogram model. Secondly, none of the patients included in this study received adjuvant immunotherapies. Thus, the possible application value of the PIIN score in predicting response to immunotherapy was not evaluated. Finally, genomic, transcriptomic, and proteomic level investigations are needed to reveal prognosis differences in patients with ICC (44, 45).

## Conclusion

The PIIN score, as a promising immune-, inflammatory-, and nutritional-related prognostic biomarker, provides insights into the biological and clinical evaluation of patients with ICC. Moreover, by integrating the PIIN scores and other clinical risk factors, the PIIN- nomograms facilitate individualized prognostic assessments for patients with ICC.

## Data availability statement

The raw data supporting the conclusions of this article will be made available by the authors, without undue reservation.

## Ethics statement

The studies involving human participants were reviewed and approved by The local Ethics Committee of West China Hospital of Sichuan University. The patients/participants provided their written informed consent to participate in this study.

## Author contributions

Conception and design: JZ, HW. Development of methodology: JZ, RH. Acquisition of data: CL, FG, TL, HL, HW. Analysis and interpretation of data: JZ, RH, DW, XF, HL. Writing, review, and/or revision of the manuscript: JZ, TL, DW, XF, HW. Administrative, technical, or material support: RH, FG, HL. Study supervision: HW. All authors read and approved the final manuscript.

## Supplementary material

Supplementary Figure 1 ROC curves showing the predictive power of the PIIN score compared to a single blood marker in the training and validation sets.
