# Supplementary figures and images for: Development and validation of a new prognostic immune–inflammatory–nutritional score for predicting outcomes after curative resection for intrahepatic cholangiocarcinoma: A multicenter study
Source: Front Immunol. 2023 Mar 31;14:1165510. doi: 10.3389/fimmu.2023.1165510 (PMC10102611; doi:10.3389/fimmu.2023.1165510)

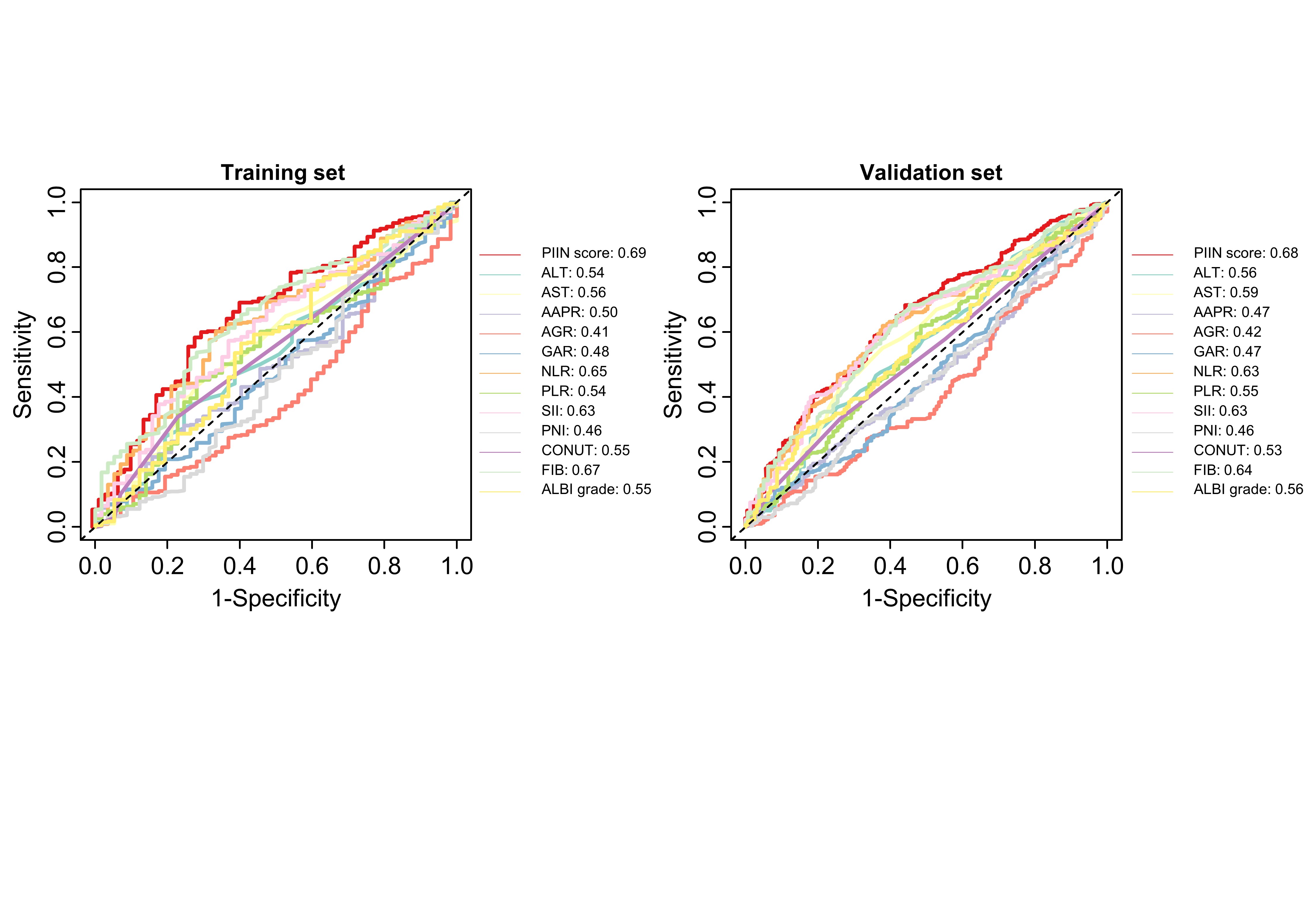

Supplement: Supplementary Figure 1 — ROC curves showing the predictive power of the PIIN score compared to a single blood marker in the training and validation sets. [file Image_1.jpeg]
