# Supplementary material for: Development and validation of a new prognostic immune–inflammatory–nutritional score for predicting outcomes after curative resection for intrahepatic cholangiocarcinoma: A multicenter study
Source: Front Immunol. 2023 Mar 31;14:1165510. doi: 10.3389/fimmu.2023.1165510 (PMC10102611; doi:10.3389/fimmu.2023.1165510)
Supplement: Supplementary file 2 [file Table_1.doc]

**Supplemental Table 1.** Assessment of undernutrition degree based on the CONUT.

| **Parameters** | **COUNT** | | | |
| --- | --- | --- | --- | --- |
|  | **Normal** | **Light** | **Moderate** | **Severe** |
| **Serum albumin (g/dL）** | ≥3.5 | 3.0-3.49 | 2.5-2.99 | ＜2.5 |
| **Score** | 1 | 2 | 4 | 6 |
| **Total lymphocyte (count/mm^3^)** | ≥1600 | 1200-1599 | 800-1199 | ＜800 |
| **Score** | 0 | 1 | 2 | 3 |
| **Total cholesterol(mg/dl)** | ＞180 | 140-180 | 100-139 | ＜100 |
| **Score** | 0 | 1 | 2 | 3 |
| **COUNT score (total)** | 0-1 | 2-4 | 5-8 | 9-12 |
| **Assessment** | Normal | Light | Moderate | Severe |

The CONUT score is calculated as the sum of the Alb score, TLC score, and T-cho score.

COUNT, controlling nutritional status; Alb: albumin; TLC: total lymphocyte; T-cho: total cholesterol
